# Supplementary material for: Development of Stilbenoid and Chalconoid Analogues as Potent Tyrosinase Modulators and Antioxidant Agents
Source: Antioxidants (Basel). 2022 Aug 17;11(8):1593. doi: 10.3390/antiox11081593 (PMC9404961; doi:10.3390/antiox11081593)
Supplement: Supplementary file 1 [file antioxidants-11-01593-s001.zip › antioxidants-1843453-supplementary.pdf]

Supplementary Materials

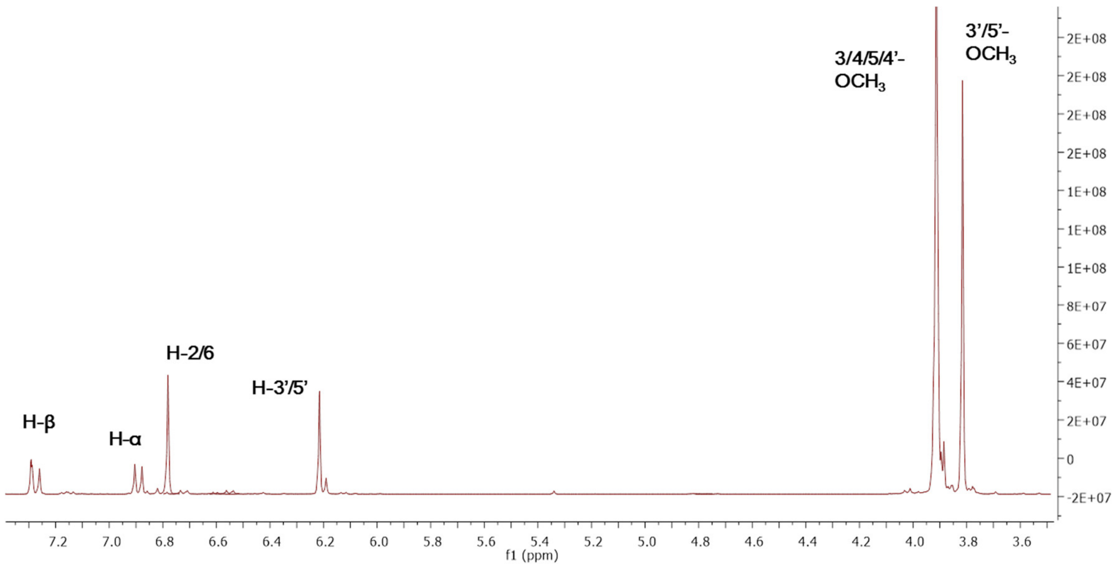

Figure S1. ^{1}H –NMR of chalcone **14**.

In the ^{1}H-NMR spectrum of compound **14** we observe the protons of the double bond of the intermediate chain (H-α and H-β) as two double peaks at δ 6.89 and 7.27 ppm, which appear deshielded as adjacent to the carbonyl group and to both aromatic rings. The coupling constant of the aforementioned protons was calculated to be 15.6 Hz, which is characteristic of the double bond trans configuration. The protons of rings A and B are coordinated as simple peaks at δ 6.79 and 6.20 ppm respectively, which intergrade for two protons each.

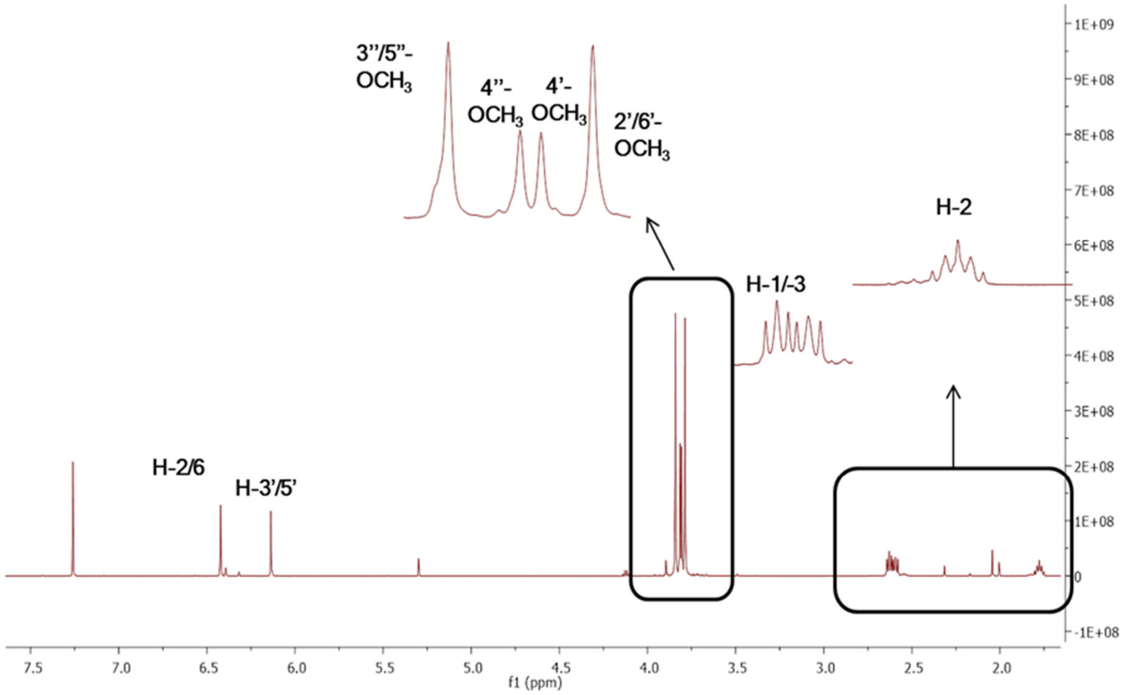

Figure S2. ^{1}H –NMR of diarylpropane **27**.

In the ^{1}H-NMR spectrum we observe some differences compared to the respective chalcone **27**. Initially, the peaks of the double bond are absent, as now the side chain consists of three methylene groups that their signals appear at 2.63, 1.77 and 1.59 ppm. The H2 "/ H-6" protons appear to more shielded fields while the methoxy groups appear to be slightly separated from each other in comparison to chalcone **27**.

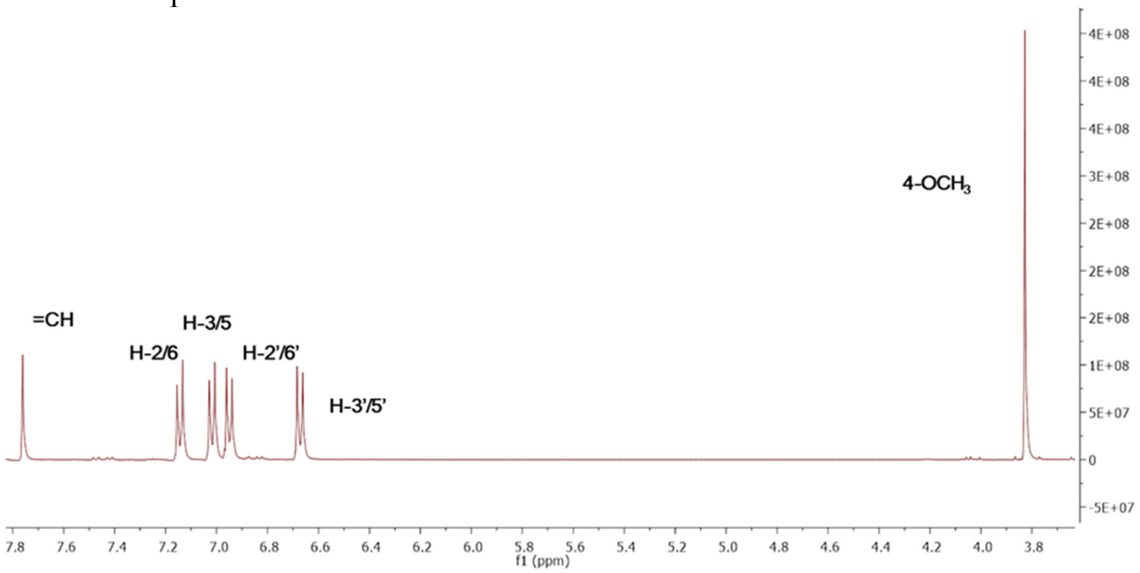

Figure S3. ^{1}H –NMR of diarylpropenoic acid **31**.

In the ^{1}H-NMR spectrum of compound **31** (SI Fig. 3), a single peak at 7.76 ppm is observed, which completes for a proton and corresponds to the olefinic proton of the intermediate chain. Based on literature data (Xiao, *et al.*, 2012), the chemical displacement of the aforementioned

proton is indicative of the configuration (E or Z) of the compound. The more deshielded chemical shift, as it was expected, are attributed to the *E* configuration (the two aromatic rings are on the same side and opposite the carboxyl group). After all, the Perkins reaction usually favors the preparation of *E*-isomers of the diphenylpropenoic acids. The condensation of the two reactants and the formation of diphenylpropenoic acid is confirmed by the HMBC spectrum, which shows the coupling of the chain olefinic proton to both the carbonyl carbonate and the C-2 'and C-6' carbons.

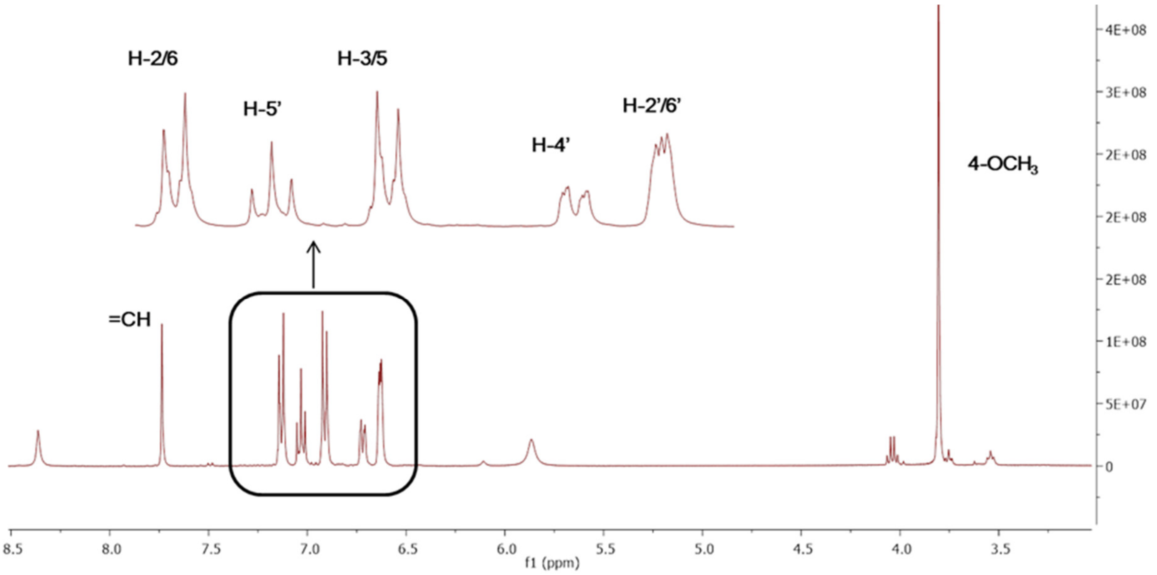

Figure S4. ^{1}H –NMR of diarylpropenoic acid **33**.

The H-5' proton appears as a triple peak with (J = 7.9 Hz), the H-4' appears as a doublet of doublets peak (J = 8.0 / 2.0 Hz), while the H-2 '/ H-6' due to chemical and magnetic equivalence appear as a multiple peak that intergrades for two protons.

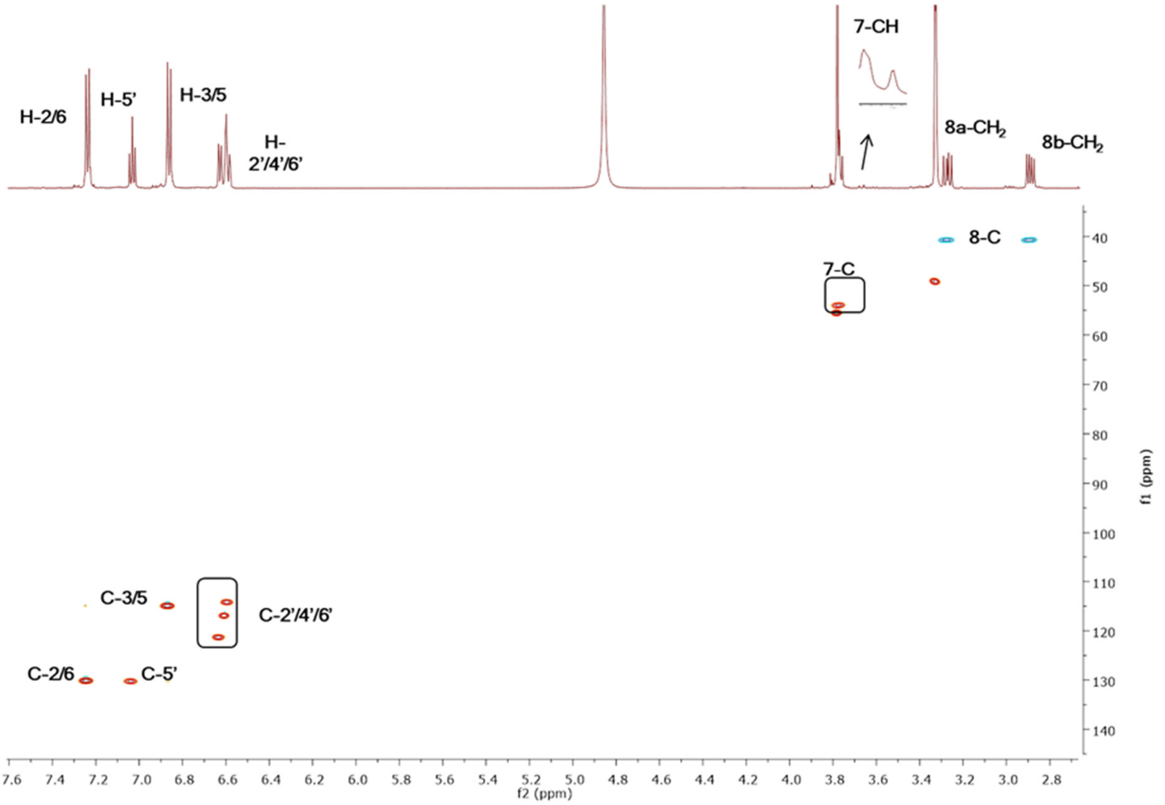

Figure S5. ^{1}H –NMR and HSQC-DEPT spectra of diarylpropanoic acid **45**.

As shown in the HSQC spectrum (SI Fig. 5), we observe the presence of methylene carbon at 40.7 ppm corresponding to the C-8 position, which is consistent with the reduction of the double bond. The protons H-8a and H-8b resonate as a double of doublet peak (J = 13.8 / 8.5 Hz and J = 13.8 / 7.0 Hz respectively), while H-7 resonates at 3.77 ppm as a triplet with J = 7.8 Hz, while the corresponding oxygenated carbon gives a peak at 54 ppm. The protons of the aromatic ring A resonate in the form of double peaks (J = 7.8 Hz) at δ 7.24 and 6.86, confirming the π-substitution in the aforementioned aromatic ring. The rotational capacity of **45** was $[\alpha]_D$=+19 and based on literature data it appears to have *S* stereochemistry (Camps, *et al.*, 1996).

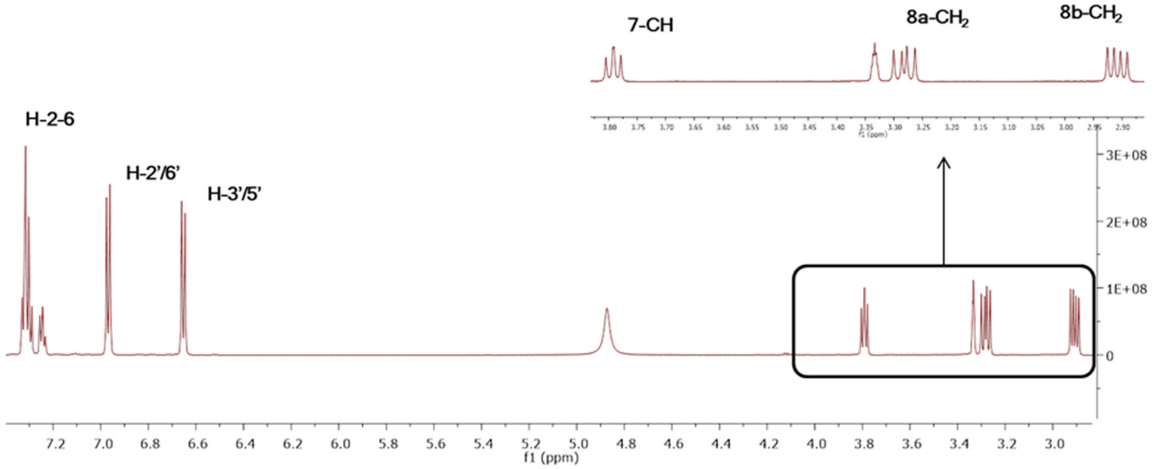

Figure S6: ^{1}H –NMR of diarylpropanoic acid **44**.

As we observed at the ^{1}H–NMR of diarylpropanoic acid **44** (SI Fig. 6) the H-7 methylene protons resonate at 3.28 and 2.91 ppm, in the form of double of doublets, and the H-8 proton to 3.79 ppm as an approximately triple peak. The protons of ring B show the expected π-substituted system, while the protons of ring A resonate at 7.33-7.24 ppm as a complex multiplet. The rotational capacity of **44** was $[\alpha]_D$ =+ 21.

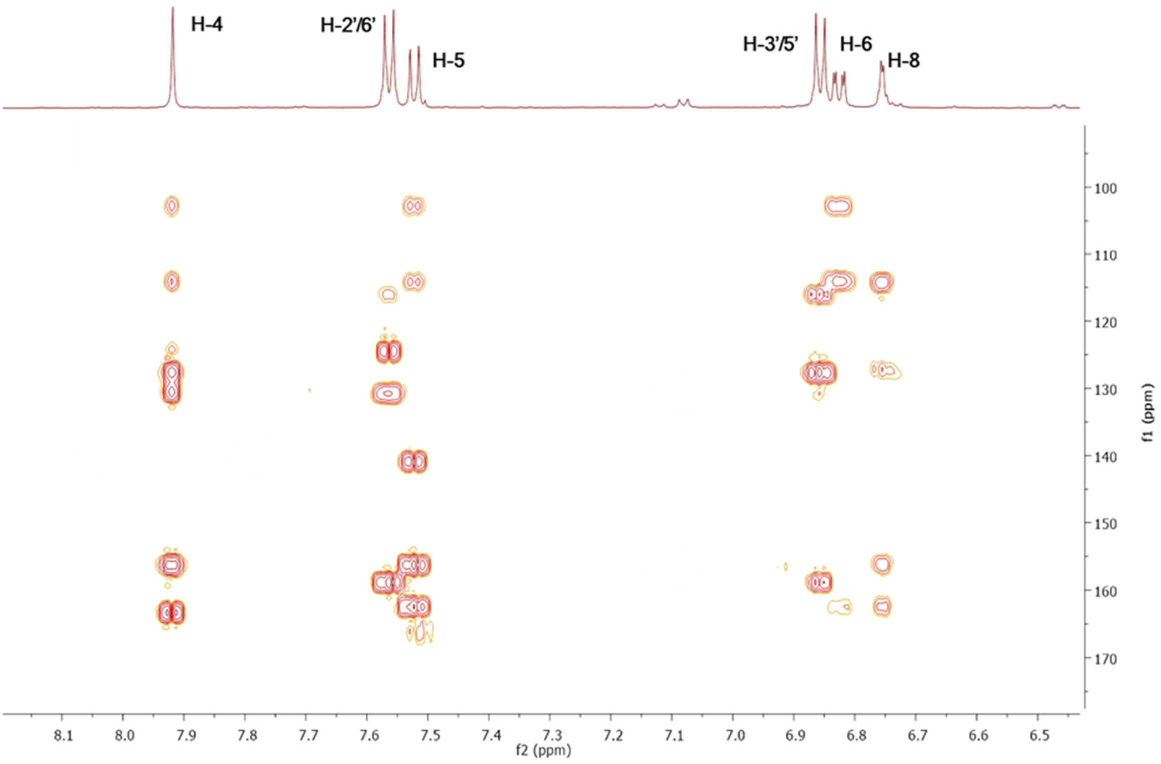

Figure S7: ^{1}H –NMR and HMBC spectra of 3-arylcoumarin **48**.

From the ^{1}H–NMR and HMBC spectra (SI Fig. 7) characteristic is the proton of the C-4 position that resonates at 7.98 ppm, i.e. in slightly lower fields compared to the case of phenylpropenoic acids. Also, proton H-4 has resonance with the oxygenated aromatic carbon at 156 ppm that corresponds to position C-8a, with which protons H-5 and H-8 also have a multiple bond correlatiot.

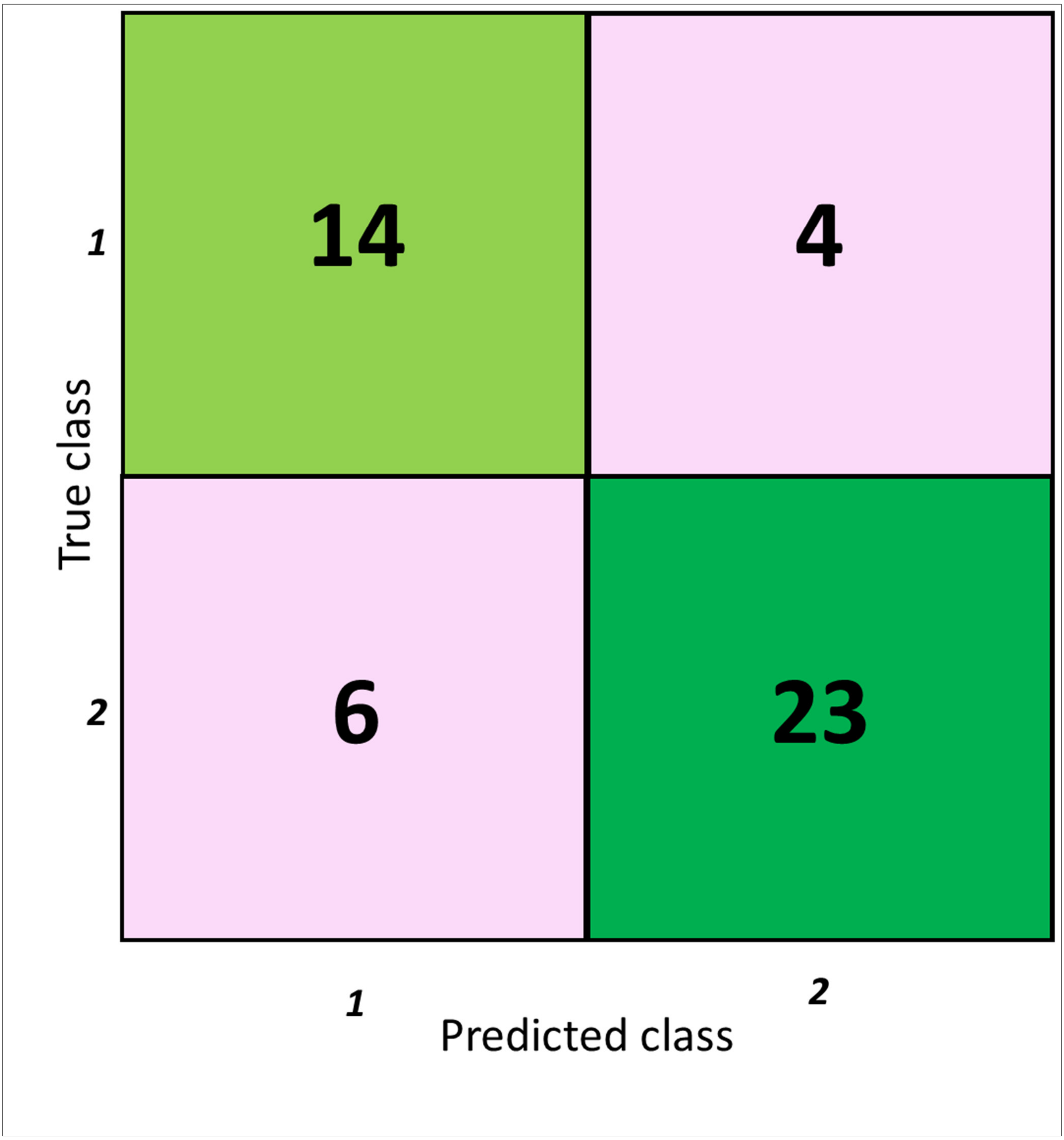

Figure S8: Confusion Matrix of Support Vector Machine classifier

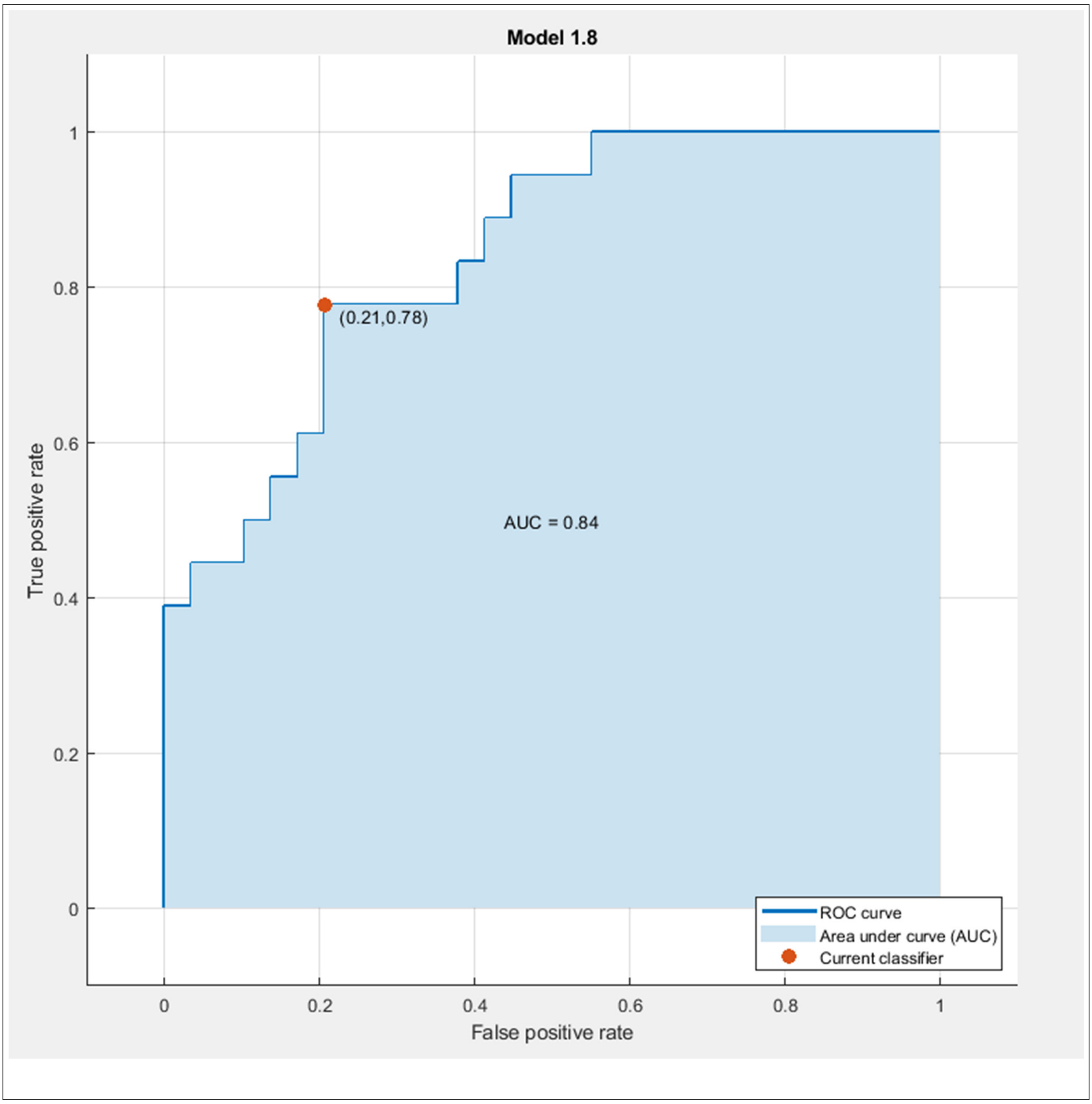

Figure S9: ROC curve of Support Vector Machine classifier.
